# Is sarcopenia an associated factor of increased administration of specific medications in patients with heart failure? A systematic review and meta-analysis

**DOI:** 10.3389/fcvm.2024.1293537

**Published:** 2024-01-25

**Authors:** Schabnam Saied, Konstantinos Prokopidis, Adeoye Adenaya, Masoud Isanejad, Rajiv Sankaranarayanan

**Affiliations:** ^1^ School of Medicine, University of Liverpool, Liverpool, United Kingdom; ^2^ Department of Musculoskeletal and Ageing Science, Institute of Life Course and Medical Sciences, University of Liverpool, Liverpool, United Kingdom; ^3^ Liverpool Centre for Cardiovascular Science, University of Liverpool, Liverpool, United Kingdom; ^4^ Liverpool University Hospitals NHS Foundation Trust, Liverpool, United Kingdom

**Keywords:** heart failure, sarcopenia, medications, skeletal muscle, drugs

## Abstract

**Background:**

There is controversy in relation to commonly used drugs in heart failure (HF) and their impact on muscle function. The aim of this study was to evaluate the odds of receiving specific medications often used in clinical practice by patients with HF and sarcopenia vs. without sarcopenia.

**Methods:**

A systematic literature search of cohort studies via databases (PubMed, Web of Science, Scopus, and Cochrane Library) was conducted from inception until March 2023. To determine if sarcopenia is linked to a higher number of specific HF-related medications, a meta-analysis using a random-effects model was used to calculate the pooled effects.

**Results:**

Our main analyses showed no significant association of sarcopenia with administration of higher HF-related medication count vs. those without sarcopenia. Those with lower appendicular lean mass (ALM) had significantly lower odds of receiving angiotensin converting enzyme inhibitors (ACE-Is)/angiotensin receptor blockers (ARBs) (OR: 0.68, 95%CI 0.50–0.90, *I*^2^ = 12%, *P* < 0.01) vs. patients with higher ALM for which age could be an important confounder based on meta-regression. No statistically significant differences were found in relation to B-blockers OR: 0.84, 95%CI 0.63–1.12, *I*^2^ = 7%, *P* = 0.24) and loop diuretics (OR: 1.19, 95%CI 0.87–1.63, *I*^2^ = 0%, *P* = 0.27). Regarding handgrip strength, gait speed, and short physical performance battery, our narrative synthesis found mixed results.

**Conclusion:**

This systematic review and meta-analysis did not find a relationship of specific medication count in sarcopenia vs. without sarcopenia in patients with HF, although increased odds of ACE-I/ARB was shown in those with higher ALM.

**Systematic Review Registration:**

PROSPERO (CRD42023411137).

## 1 Introduction

Anatomical and functional myocardial defects that impede ventricular filling or blood ejection may cause heart failure (HF). The most common cause of HF is decreased left ventricular myocardial systolic or diastolic function, but other causes include dysfunction of the valves, pericardium, or systemic conditions. HF is the most prevalent reason for hospitalisation in adults over 65, and clinically, symptoms in patients with HF are compounded by a higher prevalence of comorbidities that come with ageing (1).

It is well-established that primary sarcopenia or the loss of skeletal muscle mass and function with ageing, has a negative impact on healthspan. Secondary sarcopenia refers to the common factors outside age that could lead to losses of skeletal muscle mass and strength observed among individuals who suffer from chronic illnesses, including those with HF, contributing to increased mortality and morbidity (2). Primary and secondary sarcopenia are likely to be present together and may be additive in older people with chronic conditions, which may explain the high prevalence of this condition in patients with HF (3).

Interestingly, administration of medications has been linked to improved or impaired muscle function, depending on appropriate or inappropriate prescription, respectively. For instance, it has been suggested that angiotensin-converting enzyme inhibitors (ACE-Is) may exert positive effects on skeletal muscle in older adults, improving physical function (4) and alleviating declines in knee extension strength (5), which could be attributed to increased total insulin growth factor-1 (IGF-1) levels (6). Conversely, in a healthy older cohort (Hertfordshire Cohort Study) with a median follow-up time of 4.4 years, ACE-Is, statins, or thiazides were not associated with declines in grip strength (7), while results from the TRAIN study consisted of older people with increased cardiovascular risk also reported no significant changes in physical performance and grip strength after 6 months of fosinopril use (8). These findings may be relevant pertaining to the potential of inappropriate prescription count or duration, which may unravel potentially reduced muscle-protective responses of specific medications commonly administered in patients with HF.

The association between sarcopenia and specific drugs consumed by patients with HF has not been studied before in a systematic manner. To address this issue, the purpose of this study is to investigate observational studies in which participants with HF had sarcopenia compared to participants without sarcopenia, aiming to evaluate whether a higher prevalence of drugs commonly administered in this patient group is interlinked to sarcopenia or non-sarcopenia.

## 2 Methods

The revised 2020 Preferred Reporting Items for Systematic Reviews and Meta-Analyses (PRISMA) criteria were followed for conducting this systematic review and meta-analysis. The protocol has been entered into PROSPERO, the Prospective Register of Systematic Reviews International Database (CRD42023411137).

### 2.1 Search strategy

From the beginning until March 2023, PubMed, Scopus, Web of Science, and Cochrane Library were searched independently by K. P. and A.A. In the supporting information (Supplementary Table S1), the complete search technique and the search phrases employed are presented. A third researcher resolved any discrepancies that arose during the literature search process (M.I.).

### 2.2 Inclusion and exclusion criteria

The following criteria were used to determine which studies should be included: (i) baseline data from observational studies (i.e., cross-sectional, longitudinal, or case-control); (ii) adults aged 50 years and above with HF; (iii) clear diagnostic criteria for sarcopenia employing data from appendicular lean mass (ALM) combined with muscle strength and/or physical function outcomes; and (iv) available data from both patients with sarcopenia and without sarcopenia. Published articles were excluded if they (i) did not assess body composition with established assessment tools; ii) included patients were under the age of 50; (iii) were reviews, letters, *in vivo* or *in vitro* experiments, commentaries, or posters; and (iv) were not published as a full text and in English.

### 2.3 Data extraction

Data on the first author, publication date, country of origin, study design, participant age, left ventricular ejection fraction (LVEF) rate, number of participants, gender, reported comorbidities, assessment tool for ALM, sarcopenia definition, and type and number of HF-related medications were all extracted independently by two authors (K. P. and A.A.).

### 2.4 Risk of bias

Two independent reviewers used Newcastle–Ottawa Scale (NOS) tool to assess the risk of bias of the included studies (A.A., and S.S.). The NOS is divided into three domains: selection (4 items), comparability (1 item), and result (3 items). When a study fulfils the methodological expected standard, each item in the selection and outcome domains receives one star, with a maximum of two stars awarded for the comparability domain. Studies with a star rating from 0 to 5 have a high risk of bias, 6 to 7 a moderate risk, and 8 to 9 a low risk of bias (9).

### 2.5 Statistical analysis

To determine the odds ratio (OR) relating to the use of specific medications, quantitative data were handled as dichotomous measurements, and changes in outcomes from patients with and without sarcopenia were compared between groups. The inverse-variance approach and the random-effects model were used to determine statistical significance.

The overlap of their 95% confidence intervals (95% CI) and measures of Cochran's Q (Chi-square test) and *I*^2^ were used to analyse the statistical heterogeneity of outcome data across various studies. Low heterogeneity was defined as *I*^2^ of 30% to 49%, moderate heterogeneity as *I*^2^ of 50%–74%, and high heterogeneity as *I*^2^ of 75% and above. Sensitivity analyses that discounted the impact of sarcopenia definition that did not assess ALM and an increased risk of bias of the included cohort studies were carried out to assess the robustness of reported statistical results. The meta-analysis was synthesized using Review Manager (RevMan 5.4.1) software and a *P* value of <0.05 was considered statistically significant.

Meta-regressions were performed using a random-effects model to assess unexplained variance among studies with significant heterogeneity. Individual factors included age, LVEF (%), and body mass index (BMI), using STATA/MP 13.0.

## 3 Results

### 3.1 Literature search

**Figure 1:**
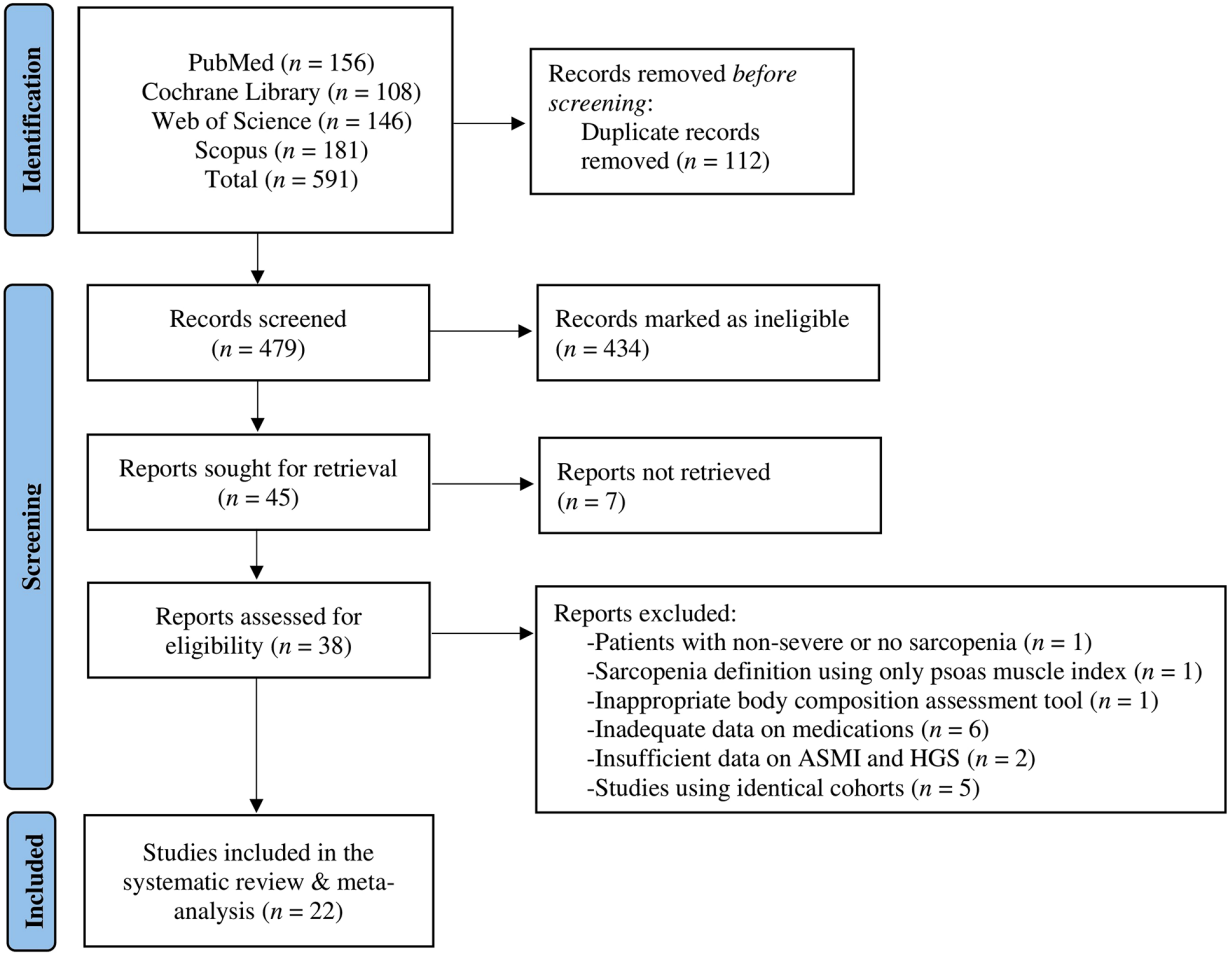
Flowchart of the employed literature search.

The initial literature search provided 591 publications. Following the exclusion of duplicates and abstracts, 38 full texts were identified as eligible for inclusion in the systematic review and meta-analysis. Of these 38 studies, six studies were dismissed due to inadequate data on listed medications, five studies because they used identical cohorts relevant to ones included in our study, two studies due to insufficient details pertinent to ALM and handgrip strength, one study used psoas muscle index as definition of sarcopenia, one study used an inappropriate equation/non-established body composition assessment tool for ALM measurements, and one study included patients with non-severe or no sarcopenia. In total, 22 studies (10–31) were included in the systematic review and meta-analysis exploring the association of different HF-related medications with sarcopenia vs. without sarcopenia in cohorts with patients with HF (Figure 1). Characteristics of the included studies are summarised in Table 1.

**Table 1:** Study and participant characteristics of the included studies in the systematic review and meta-analysis.

Study Year Country	Sarcopenia or muscle dysfunction definition	Total *n* (M/F)	HF with sarcopenia or muscle dysfunction			HF without sarcopenia or muscle dysfunction			Reported comorbidities	Body composition assessment tool
			*n* (M/F)	Age (SD)	LVEF (%)	*n* (M/F)	Age (SD)	LVEF (%)		
Bieger 2023 Brazil	EWGSOP2	106 (71/35)	25 (18/7)	73.1 ± 8.1	92% of participants were <40%	81 (53/28)	67.8 ± 6.1	64.2% of participants were <40%	T2D, CKD, AF, Stroke/AMI, DLP, Systemic Arterial HT, CAD	BIA
Peng 2023 China	AWGS 2014	62 (37/25)	29 (13/16)	75.1 ± 8.2	55 (38, 60)	33 (24/9)	71.8 ± 7.9	57 (39.5, 61.5)	–	BIA
Saito 2022a Japan	AWGS 2014	575 (319/256)	119 (81/38)	82 (76, 86)	45 ± 18	456 (238/218)	81 (74, 86)	46 ± 16	T2D, AF, CAD, COPD, HT	BIA
Valdiviesso 2022 Portugal	EWGSOP2	136 (90/46)	25 (3/22)	67.0 (52, 70.5)	42.3 ± 16.5	111 (87/24)	58 (49, 67)	36.8 ± 12.9	AMI, AF, T2D	MAMC
Eschalier 2021 France	EWGSOP1	140 (82/58)	91 (54/37)	78.2 ± 9.0	42.8 ± 14.7	49 (28/21)	71.4 ± 10.9	40.7 ± 14.0	T2D, CKD, AF, DLP, HT, VA	BIA
Fonseca 2020 Brazil	EWGSOP1	168 (168/0)	66 (66/0)	60 (55, 63)	25 (21, 34)	102 (102/0)	56 (50, 61)	27 (22, 33)	–	DXA
Kono 2020 Japan	Japanese Geriatrics Society	186 (81/105)	77 (15/62)	85.6 ± 6.9	62.0 ± 16.1	109 (66/43)	75.3 ± 9.0	45.8 ± 17.7	T2D, COPD, Stroke, HT	–
Ogawa 2020 Japan	AWGS 2014	100 (62/38)	47 (25/22)	80 (75, 84)	48 (30, 66)	53 (37/16)	75 (68, 79.5)	40.5 (30, 60.8)	Stroke, AF, T2D, DLP, HT	BIA
Harada 2017 Japan	AWGS 2014	322 (187/135)	90 (34/56)	78 ± 8	All: 61.1 ± 12.8	232 (153/79)	69 ± 13	All: 61.1 ± 12.8	Dyslipidemia, Stroke, Obesity, T2D, CKD, PAD	
Onoue 2016 Japan	Ishii Index	119 (73/46)	82 (53/29)	77.6 ± 5.4	53.8 ± 12.3	37 (20/17)	72.0 ± 5.9	58.8 ± 11.8	T2D, DLP, HT	–
Katano 2022 Japan	ASMI (≤7.00 kg/m^2^ for males; ≤ 5.40 kg/m^2^ for females)	539 (307/232)	335 (201/134)	73 (66, 81)	45.9 (32.4, 62.0)	204 (106/98)	72 (60, 82)	51.7 (36.2, 64.0)	T2D, HT, DLP, Cancer, AF	DXA
Saito 2022b Japan	ASMI (≤7.00 kg/m^2^ for males; ≤5.40 kg/m^2^ for females)	226 (117/109)	120 (85/35)	82.1 ± 7.0	47 ± 17	106 (32/74)	79.8 ± 8.3	48 ± 16	T2D, HT, COPD	DXA
Sato 2020 Japan	ASMI (≤7.00 kg/m^2^ for males; ≤5.40 kg/m^2^ for females)	387 (315/72)	97 (79/18)	74 ± 9	43 ± 12	290 (236/54)	63 ± 13	47 ± 11	T2D, HT, DLP	DXA
Emami 2018 Germany	ASMI <7.26 kg/m^2^ for males	168 (168/0)	30 (30/0)	73.1 ± 8.4	36.7 ± 12.4	138 (138/0)	66.4 ± 10.8	38.7 ± 12.8	T2D, HT, CKD, DLP	DXA
Tsuchida 2018 Japan	ASMI (<6.87 kg/m^2^ for males; <5.46 kg/m^2^ for females)	38 (25/13)	20 (16/4)	77.9 ± 9.1	45.6 ± 13.8	18 (9/9)	72.0 ± 13.1	49.6 ± 16.9	T2D, HT, COPD, AF	DXA
Castillo-Martinez 2020 Mexico	HGS (<10.1 kg/m^2^ for males; <7.95 kg/m^2^ for females)	336	164	M: 64.9 ± 16.3 F: 63.4 ± 16.9	M: 44 ± 17 F: 47 ± 17	172	M: 56.6 ± 15.5 F: 55.7 ± 17.8	M: 45 ± 15 F: 49 ± 16	T2D, HT, CKD	–
Chung 2014 USA	HGS <25% bodyweight vs. ≥25% bodyweight	72 (64/8)	16 (12/4)	61 ± 3	20.3 ± 1.5	56 (52/4)	59 ± 2	17.3 ± 0.6	–	–
Ozawa (Kitasato) 2021 Japan	Gait Speed (Slow SGS ratio <0.527 vs. Non-slow SGS ratio ≥0.527)	1,247 (724/523)	213 (109/104)	78 (74, 84)	46 ± 17	1,034 (615/419)	75 (71, 81)	47 ± 17	T2D, HT, AF, COPD	–
Ozawa (FRAGILE-HF) 2021 Japan	Gait Speed (Slow SGS ratio <0.527 vs. Non-slow SGS ratio ≥0.527)	1,301 (740/561)	358 (184/174)	82 (77, 87)	48 ± 17	943 (556/387)	80 (73, 86)	45 ± 17	T2D, HT, AF, COPD	–
Pulignano 2020 Italy	Gait Speed (Tertiles) (≤0.65 m/s vs. ≥ 1.0 m/s)	203 (113/90)	115 (56/59)	80.2 ± 5.6	36.4 ± 12.7	88 (57/31)	76.4 ± 4.8	35.3 ± 9.8	T2D, HT, AF, COPD	–
Chiaranda 2013 Italy	Gait Speed (Quartiles)	642 (642/0)	316 (316/0)	65 ± 9	53 ± 11	326 (326/0)	57 ± 9	58 ± 10	–	–
Matsuzawa 2013 Japan	Gait Speed (Tertiles)	313 (257/56)	158 (130/28)	69.9 ± 10.8	50.3 ± 13.0	155 (127/28)	58.5 ± 10.4	56.4 ± 9.8	T2D, HT, DLP	–
Kitai 2021 Japan	SPPB (<7 vs. ≥ 7)	1,192 (682/510)	373 (154/219)	85 (80, 89)	All: 45% (32, 60)	819 (528/291)	79 (72, 84)	All: 45% (32, 60)	T2D, COPD, AF, CAD, HT	–

AF, atrial fibrillation; AMI, acute myocardial infarction; ASMI, appendicular skeletal muscle index; AWGS, Asian Working Group for Sarcopenia; BIA, bioelectrical impedance; CAD, coronary artery disease; CKD, chronic kidney disease; COPD, chronic obstructive pulmonary disease; DLP, dyslipidemia; DXA, dual x-ray absorptiometry; EWGSOP, European Working Group on Sarcopenia in Older People; F, females; HGS, handgrip strength; HT, hypertension; LVEF, left ventricular ejection fraction; M, males; MAMC, mid-upper arm muscle circumference; PAD, peripheral artery disease; SD, standard deviation; SGS, slow gait speed; SPPB, short physical performance battery; T2D, type 2 diabetes; VA, Vascular Arteriopathy.

Data are expressed as mean ± SD.

Data are expressed as median (IQR).

### 3.2 Descriptive results

Ten studies assessed the prevalence of different HF-related medications in patients with sarcopenia (12, 13, 16, 18, 20, 23, 24, 26, 27, 31), five studies in patients with low ALM (11, 14, 17, 25, 29), two studies with low handgrip strength (21, 30), four studies with low gait speed (10, 15, 22, 28), and one study with low short physical performance battery (SPPB) scores (19). Detailed characteristics of the included studies are outlined in Table 1.

### 3.3 Definition of sarcopenia

To define sarcopenia, two studies used the European Working Group on Sarcopenia in Older People 2 (EWGSOP2) criteria (13, 27), two studies used the EWGSOP1 criteria (20, 24), four studies used the Asian Working Group for Sarcopenia 2014 criteria (18, 23, 26, 31), one study used the Japanese Geriatrics Society criteria (16), and one study used the Ishii index (12).

### 3.4 Prevalence of different medications in patients with HF and sarcopenia vs. without sarcopenia

**Figure 2:**
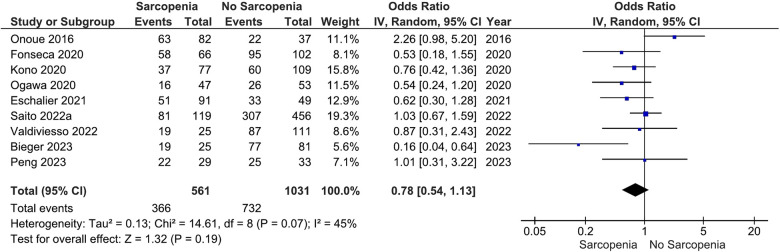
Association of ACE-I/ARB administration in patients with HF and sarcopenia versus without sarcopenia.

**Figure 3:**
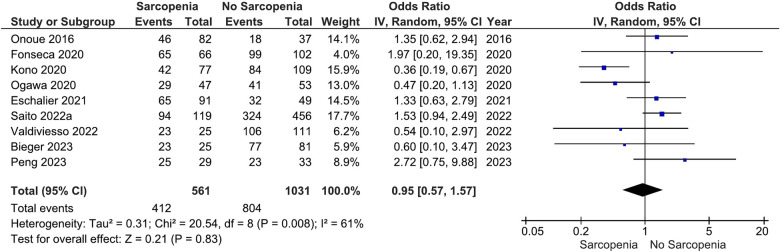
Association of B-blocker administration in patients with HF and sarcopenia versus without sarcopenia.

**Figure 4:**
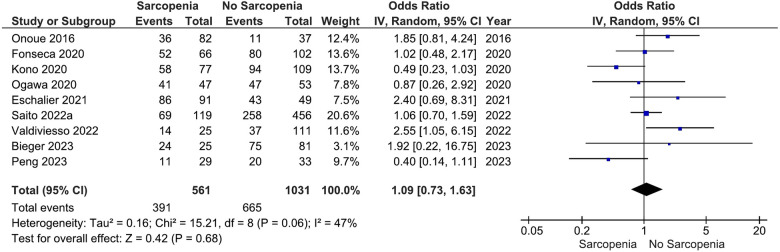
Association of loop diuretic administration in patients with HF and sarcopenia versus without sarcopenia.

**Figure 5:**
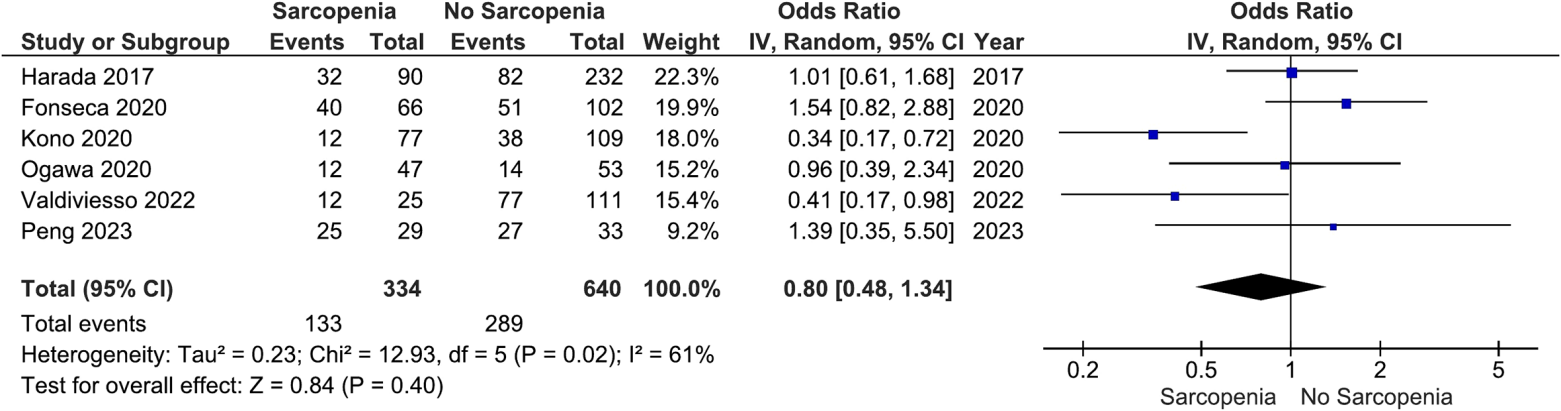
Association of statin administration in patients with HF and sarcopenia versus without sarcopenia.

Our main analysis showed no significant association of sarcopenia with angiotensin converting enzyme inhibitors (ACE-Is)/angiotensin receptor blockers (ARBs) use vs. no sarcopenia (OR: 0.78, 95%CI 0.54–1.13, *I*^2^ = 45%, *P* = 0.19) (Figure 2). Likewise, no differences were found in relation to B-blocker use (OR: 0.95, 95%CI 0.57–1.57, *I*^2^ = 61%, *P* = 0.83) (Figure 3), loop diuretics (OR: 1.09, 95%CI 0.73–1.63, *I*^2^ = 47%, *P* = 0.68) (Figure 4), and statins (Sarcopenia, *n* = 334; No sarcopenia, *n* = 640; OR: 0.80, 95%CI 0.48–1.34, *I*^2^ = 61%, *P* = 0.40) (Figure 5).

Sensitivity analyses based on impartial sarcopenia definition did not reveal any significant differences (ACE-I/ARB; OR: 0.65, 95%CI 0.42–1.02, *I*^2^ = 41%, *P* = 0.06 (Figure S1); B-blockers; OR: 1.19, 95%CI 0.73–1.95, *I*^2^ = 35%, *P* = 0.49 (Figure S2); Loop diuretics; OR: 1.00, 95%CI 0.70–1.43, *I*^2^ = 9%, *P* = 0.98 (Figure S3); Statins; OR: 1.16, 95%CI 0.82–1.65, *I*^2^ = 0%, *P* = 0.39 (Figure S4)) nor by excluding studies with high risk of bias (ACE-I/ARB; OR: 0.81, 95%CI 0.38–1.73, *I*^2^ = 75%, *P* = 0.58 (Figure S5); B-blockers; OR: 1.39; 95%CI 0.98–1.98, *I*^2^ = 0%, *P* = 0.07 (Figure S6); Loop diuretics; OR: 1.26, 95%CI 0.89–1.79, *I*^2^ = 0%, *P* = 0.19 (Figure S7)). No sensitivity analysis was conducted regarding statins given that all studies were scored as high risk of bias.

### 3.5 Prevalence of different medications in patients with HF and low ALM vs. higher ALM

**Figure 6:**
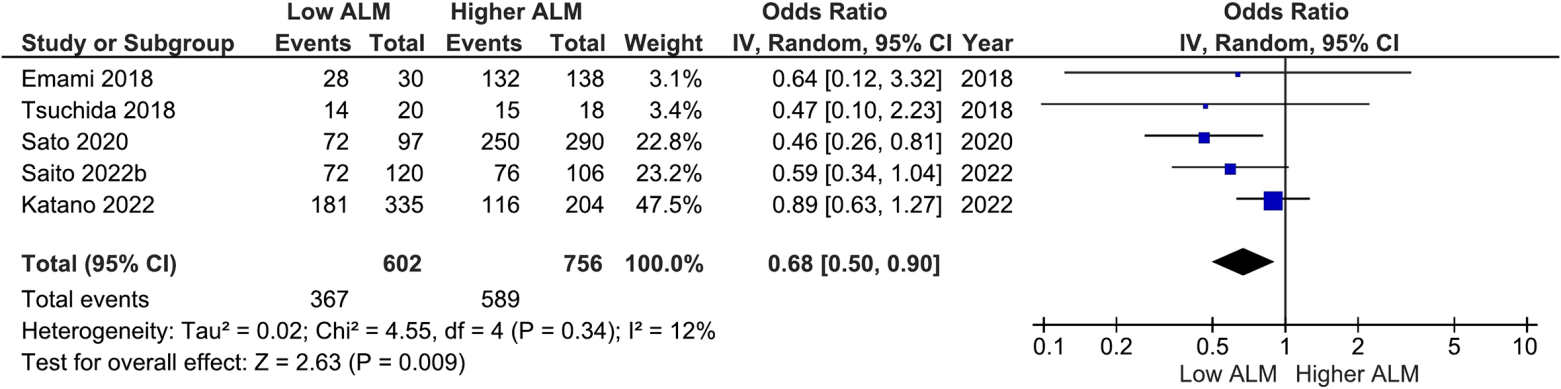
Association of ACE-I/ARB administration in patients with HF and higher ALM versus low ALM.

**Figure 7:**
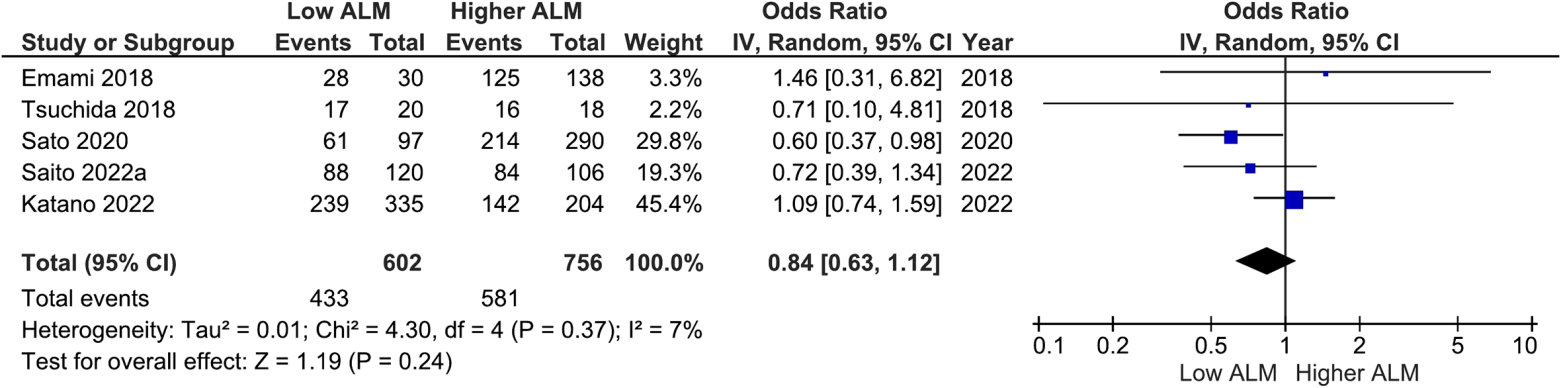
Association of B-blocker administration in patients with HF and higher ALM versus low ALM.

**Figure 8:**
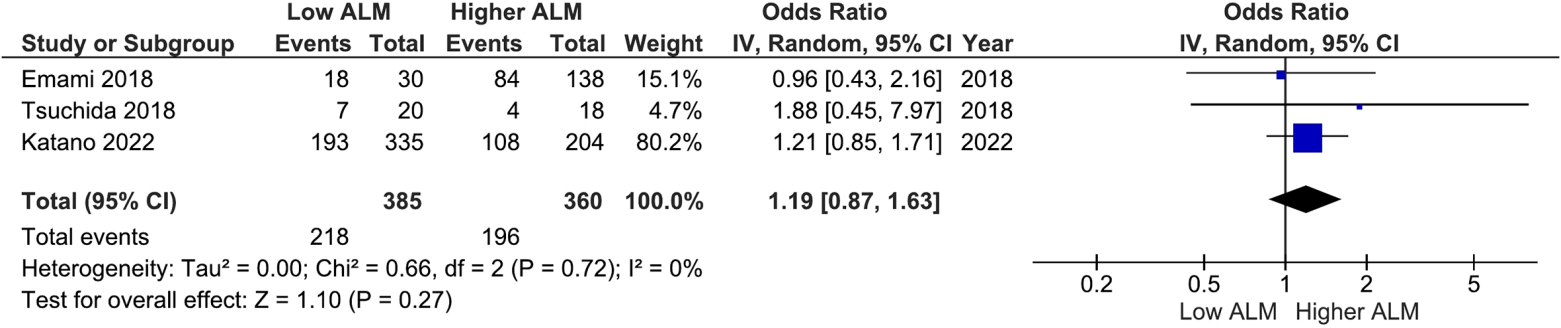
Association of loop diuretics administration in patients with HF and higher ALM versus low ALM.

Our main analysis found significantly lower odds of ACE-I/ARB (OR: 0.68, 95%CI 0.50–0.90, *I*^2^ = 12%, *P* < 0.01) (Figure 6) in patients with lower vs. higher ALM. No statistically significant differences were found in relation to B-blockers (OR: 0.84, 95%CI 0.63–1.12, *I*^2^ = 7%, *P* = 0.24) (Figure 7) and loop diuretics (OR: 1.19, 95%CI 0.87–1.63, *I*^2^ = 0%, *P* = 0.27) (Figure 8). A higher prevalence between statins and low ALM was found in one study (11), however, it was not considered significant (*P* = 0.33).

Sensitivity analyses based on exclusion of studies with increased risk of bias did not alter the findings of the main analyses [ACE-I/ARB; OR: 0.66, 95%CI 0.44–0.99, *I*^2^ = 53%, *P* = 0.04 (Figure S8); B-blockers; OR: 0.81, 95%CI 0.55–1.19, *I*^2^ = 47%, *P* = 0.27 (Figure S9)].

### 3.6 Prevalence of different medications in patients with HF and low handgrip strength vs. higher handgrip strength

In one study, no significant differences were found between low and higher handgrip strength index groups, and B-blockers (30). A higher % of patients with low handgrip strength was receiving statins (63% vs. 43%) and loop diuretics (88% vs. 82%), while those with higher handgrip strength were administered a greater proportion of ACE-I/ARBs (54% vs. 44%) (21).

### 3.7 Prevalence of different medications in patients with HF and low gait speed vs. higher gait speed

In the Kitasato cohort from the study by Ozawa et al. (2021), no differences were found between slow gait and faster gait groups in relation to ACE-I/ARB, B-blocker, MRA, and loop diuretics (15). The slow gait group however in the FRAGILE-HF cohort had a lower prevalence of B-blocker administration vs. the non-slow group (67.3% vs. 74.9%, *P* < 0.01). Those with increased gait speed also exhibited higher prevalence of B-blocker administration vs. the slowest tertile group (65.9% vs. 49.6%, *P* = 0.04) with no differences related to ACE-I/ARBs (22). No changes among gait speed quartiles regarding ACE-I/ARBs, and B-blockers were observed, however, those with slow walking speed exhibited a higher prevalence of loop diuretics vs. faster groups (Quartile I: 26.6% vs. Quartile IV: 10.4%, *P* < 0.05). Similarly, those with slower walking speed were in a greater proportion in receiving statins (Quartile I: 50.3% vs. Quartile IV: 60.1%, *P* < 0.05) (10). Finally, Matsuzawa et al. (2013) found a higher prevalence of ACE-I/ARBs in the fastest tertile vs. the slowest tertile group (89.7% vs. 77.2%, *P* < 0.05), although no changes were highlighted in terms of B-blockers (57.4% vs. 51.9%, *P* > 0.05).

### 3.8 Prevalence of different medications in patients with HF and low SPPB vs. higher SPPB

Only one study was included in this systematic review pertinent to SPPB scores (19). Those with a higher score (≥7) had a significantly higher prevalence of B-blockers (76.8% vs. 68.9%, *P* < 0.01), ACE-I/ARBs (71.6% vs. 61.4%, *P* < 0.01), and direct oral anticoagulants (35% vs. 29%), but not in relation to MRAs (9.2% vs. 6.7%), digoxin (3.4% vs. 1.6%), and warfarin (24% vs. 23%). Interestingly, those with a higher score had a lower prevalence of loop diuretic use (86% vs. 92%, *P* < 0.01).

### 3.9 Meta-regression analyses

The increased heterogeneity displayed for the prevalence of higher number of HF-related medications in patients with vs. without sarcopenia was further investigated through meta-regression analyses, using age, LVEF, and BMI as covariates. It was found that age, LVEF, and BMI were significant moderators of B-blockers, BMI of loop diuretics, and LVEF of statins, in patients with sarcopenia vs. without sarcopenia (Table S2). In addition, age was a significant moderator of ACE-I/ARB and B-blocker count in patients with lower vs. higher ALM (Table S3).

### 3.10 Risk of bias assessment

The overall quality of the included studies was considered moderate. In particular, four studies had a low risk of bias (15, 17, 18, 30), 10 studies had a moderate risk (10, 12, 14, 19–22, 27–29), while eight studies had a high risk of bias (11, 13, 16, 23–26, 31). A detailed description of the risk of bias is shown in Table S4.

## 4 Discussion

In this systematic review and meta-analysis, we found no differences in specific drug administration prevalence in subjects with HF and sarcopenia vs. without sarcopenia. When we attempted to evaluate the impact of individual sarcopenia components, our analysis revealed significantly higher odds of ACE-I/ARB administration in patients with higher vs. lower ALM. In relation to handgrip strength, gait speed, and SPPB status, our narrative synthesis found mixed results that do not allow the extrapolation of conclusions, confidently. It is worth noting that age was a significant moderator of ACE-I/ARB count, which could explain, in part, our statistically significant findings.

Cross-sectional studies have shown a positive link among ACE-I/ARB usage, ALM, and muscle function (32, 33), while others have not observed such relationship (34). Likewise, longitudinal and clinical studies have failed to report positive outcomes in relation to muscle strength and physical performance (7, 8). A recent study showed that losartan could enhance the effects of exercise on muscle mass and muscle cross-sectional area in mice (35), however, in community-dwelling older adults and older subjects with chronic obstructive pulmonary disease (COPD), ACE-Is did not show benefits in response to an exercise programme (36, 37). Currently, research is lacking in patients with HF in order to show how ACE-I/ARBs could be connected to greater ALM.

Recently, an observational study linked the combination of ARBs and statin with higher ALM in patients with cardiovascular disease (38), however, research around the impact of statins on skeletal muscle is controversial. Mechanistic studies conducted in rats have shown that statins could induce acute muscle damage (39), although in mdx mice with Duchenne muscular dystrophy, no signs of inflammation, fibrosis, and angiogenesis reflecting muscle injury were observed (40). Furthermore, *dmd/mdx* mice treated with simvastatin have displayed decreased CYBB/NOX2-mediated oxidative stress and higher autophagy that corresponded with reduced muscle damage and inflammation, and increased muscle force production (41). Conversely, in C2C12 mice myotubes, simvastatin administration led to overexpression of myostatin in skeletal muscle (42), while in human myotubes, it was linked with impaired adenosine diphosphate (ADP)-stimulated maximal mitochondrial respiratory capacity and mitochondrial oxidative stress (43). Although some mechanistic evidence primarily from animal and cell models indicate a negative response of skeletal muscle to statin administration, these findings are currently unknown in humans and particularly patients with HF. These results also confirm our non-significant association of statin administration count in sarcopenia vs. no sarcopenia. Nevertheless, considering the various cardiovascular benefits of statins, future research unravelling its impact on skeletal muscle may be critical.

### 4.1 Strengths and limitations

This is the first study attempting to quantify the relationship between sarcopenia and its parameters in patients with HF with specific drug administration. One of the limitations of this study is the possibility of reverse causation pertinent to those with higher ALM to be receiving more ACE-I/ARBs on the actual impact of these medications in promoting better muscle health. The nature of this cross-sectional study is unable or provide definitive answers and considering the limited research around this area in patients with HF, accurate conclusions cannot be extrapolated. In addition, some studies used different definitions of sarcopenia alongside different body composition assessment tools which could explain, in part, the moderate heterogeneity among studies in our analyses. Furthermore, considering that the majority of studies were conducted in Japan, our findings do not represent the general patient with HF and sarcopenia and lower ALM. Angiotensin receptor neprilysin inhibitor and sodium-glucose co-transporter-2 (SGLT2) inhibitors are relatively new medications in use for HF and there is therefore a relative paucity of studies that have looked at the relationship of sarcopenia and use of these medications. Lastly, it is worth noting that there is a likelihood of inflation in the number of listed medications, which could misrepresent their status, given the inaccuracies that may occur due to faulty coding of drug prescriptions and/or incorrect tabulations performed electronically.

## 5 Conclusions

This systematic review and meta-analysis found no link between number of specific drug administration in patients with HF and sarcopenia vs. without sarcopenia, although increased odds of ACE-I/ARB prescription was found in those with higher ALM. The emergence of inappropriate prescription is a critical phenomenon in medicine, impacting patient healthcare and potentially musculoskeletal health. Future research in patients with HF could clarify whether specific medications are linked to muscle-protective or impairing properties and identify potential inappropriate medications.

## Data Availability

The original contributions presented in the study are included in the article/Supplementary Material, further inquiries can be directed to the corresponding author.

## Supplementary material

Supplementary Figure S1 Association of ACE-I/ARB administration in patients with HF and sarcopenia versus without sarcopenia according to sarcopenia definition.

Supplementary Figure S2 Association of B-blocker administration in patients with HF and sarcopenia versus without sarcopenia according to sarcopenia definition.

Supplementary Figure S3 Association of loop diuretic administration in patients with HF and sarcopenia versus without sarcopenia according to sarcopenia definition.

Supplementary Figure S4 Association of statin administration in patients with HF and sarcopenia versus without sarcopenia according to sarcopenia definition.

Supplementary Figure S5 Association of ACE-I/ARB administration in patients with HF and sarcopenia versus without sarcopenia based on risk of bias.

Supplementary Figure S6 Association of B-blockers administration in patients with HF and sarcopenia versus without sarcopenia based on risk of bias.

Supplementary Figure S7 Association of loop diuretic administration in patients with HF and sarcopenia versus without sarcopenia based on risk of bias.

Supplementary Figure S8 Association of ACE-I/ARB administration in patients with HF and higher ALM versus low ALM based on risk of bias.

Supplementary Figure S9 Association of B-blockers administration in patients with HF and higher ALM versus low ALM based on risk of bias.
