# Supplementary material for: Is sarcopenia an associated factor of increased administration of specific medications in patients with heart failure? A systematic review and meta-analysis
Source: Front Cardiovasc Med. 2024 Jan 25;11:1293537. doi: 10.3389/fcvm.2024.1293537 (PMC10850377; doi:10.3389/fcvm.2024.1293537)
Supplement: Supplementary file 1 [file Table1.docx]

**Table S1.**Search terms employed in the screening based on title, abstract, and keywords in the literature search.

| **Database** | **Search terms** |
| --- | --- |
|  |  |
| PubMed | heart failure AND (“polypharmacy” OR “prescription*” OR “number of prescriptions” OR “multiple prescriptions”  OR “drug*” OR “numbers of drugs” OR "multiple drugs" OR “medication*” OR "multiple medications")  AND ("sarcopeni*" OR “gait speed” OR “walking speed” OR “handgrip strength” OR “grip strength” OR “SPPB” OR  Short physical performance battery OR appendicular lean mass OR appendicular skeletal muscle OR “ASMI”) |
| Cochrane Library | (heart failure AND (“polypharmacy” OR “prescription*” OR “number of prescriptions” OR “multiple prescriptions”  OR “drug*” OR “numbers of drugs” OR "multiple drugs" OR “medication*” OR "multiple medications")  AND ("sarcopeni*" OR “gait speed” OR “walking speed” OR “handgrip strength” OR “grip strength” OR “SPPB” OR  Short physical performance battery OR appendicular lean mass OR appendicular skeletal muscle OR “ASMI”) |
| Web of Science | heart failure AND (“polypharmacy” OR “prescription*” OR “number of prescriptions” OR “multiple prescriptions”  OR “drug*” OR “numbers of drugs” OR "multiple drugs" OR “medication*” OR "multiple medications")  AND ("sarcopeni*" OR “gait speed” OR “walking speed” OR “handgrip strength” OR “grip strength” OR “SPPB” OR  Short physical performance battery OR appendicular lean mass OR appendicular skeletal muscle OR “ASMI”) |
| Scopus | heart AND failure AND sarcopenia OR muscle AND strength OR handgrip AND strength OR gait AND speed OR  muscle AND mass AND polypharmacy OR drugs OR medications |
