# Supplementary material for: Is sarcopenia an associated factor of increased administration of specific medications in patients with heart failure? A systematic review and meta-analysis
Source: Front Cardiovasc Med. 2024 Jan 25;11:1293537. doi: 10.3389/fcvm.2024.1293537 (PMC10850377; doi:10.3389/fcvm.2024.1293537)
Supplement: Supplementary file 2 [file Table2.docx]

**Table S2.** Meta-regression analysis of patients with HF and sarcopenia vs. no sarcopenia.

| **Drug** | *r* | SE | 95%CI | *z* | p |
| --- | --- | --- | --- | --- | --- |
| *ACE-I/ARBs* |  |  |  |  |  |
| Age | -0.0294 | 0.0355 | -0.10, 0.04 | -0.83 | 0.41 |
| LVEF | -0.054 | 0.0193 | -0.04, 0.03 | -0.28 | 0.78 |
| BMI | -0.523 | 0.1785 | -0.40, 0.03 | -0.30 | 0.77 |
| *B-blockers* |  | | | | |
| Age | -0.1339 | 0.0355 | -0.20, -0.06 | -3.77 | <0.01* |
| LVEF | -0.058 | 0.0148 | -0.09, -0.03 | -3.93 | <0.01* |
| BMI | 0.856 | 0.2693 | 0.33, 1.38 | 3.18 | <0.01* |
| *Loop diuretics* |  | | | | |
| Age | -0.057 | 0.058 | -0.17, 0.06 | -0.98 | 0.33 |
| LVEF | -0.026 | 0.029 | -0.08, 0.03 | -0.91 | 0.36 |
| BMI | 0.472 | 0.223 | 0.03, 0.91 | 2.11 | 0.04* |
| *Statins* |  | | | | |
| Age | -0.158 | 0.095 | -0.34, 0.03 | -1.67 | 0.10 |
| LVEF | -0.070 | 0.034 | -0.14, -0.00 | -2.06 | 0.04* |
| BMI | 0.174 | 0.355 | -0.52, 0.87 | 0.49 | 0.62 |

*Indicates p<0.05
