# Supplementary material for: Is sarcopenia an associated factor of increased administration of specific medications in patients with heart failure? A systematic review and meta-analysis
Source: Front Cardiovasc Med. 2024 Jan 25;11:1293537. doi: 10.3389/fcvm.2024.1293537 (PMC10850377; doi:10.3389/fcvm.2024.1293537)
Supplement: Supplementary file 3 [file Table3.docx]

**Table S3.** Meta-regression analysis of patients with HF and lower ALM vs. higher ALM.

| **Drug** | *r* | SE | 95%CI | *z* | p |
| --- | --- | --- | --- | --- | --- |
| *ACE-I/ARBs* |  |  |  |  |  |
| Age | -0.073 | 0.0238 | -0.12, -0.03 | -3.04 | 0.002* |
| LVEF | 0.133 | 0.4812 | -0.81, 1.08 | 0.28 | 0.78 |
| BMI | 0.433 | 0.6081 | -0.76, 1.62 | 0.71 | 0.48 |
| *B-blockers* |  | | | | |
| Age | -0.066 | 0.0296 | -0.12, -0.01 | -2.24 | 0.03* |
| LVEF | 0.707 | 0.8297 | -0.92, 2.33 | 0.85 | 0.39 |
| BMI | 0.558 | 1.030 | -1.46, 2.58 | 0.54 | 0.59 |
| *Loop diuretics* |  | | | | |
| Age | -0.044 | 0.104 | -0.25, 0.16 | -0.42 | 0.67 |
| LVEF | -0.133 | 0.273 | -0.67, 0.40 | -0.49 | 0.63 |
| BMI | 0.235 | 1.001 | -1.73, 2.20 | 0.23 | 0.81 |

*Indicates p<0.05
