# Supplementary material for: Is sarcopenia an associated factor of increased administration of specific medications in patients with heart failure? A systematic review and meta-analysis
Source: Front Cardiovasc Med. 2024 Jan 25;11:1293537. doi: 10.3389/fcvm.2024.1293537 (PMC10850377; doi:10.3389/fcvm.2024.1293537)
Supplement: Supplementary file 4 [file Table4.docx]

**Table S4.** Quality assessment of the included studies based on the Newcastle-Ottawa Quality Assessment Form for Cohort Studies tool.

| Reference | Selection | | | | Comparability | | Outcome | | | Overall cumulative score |
| --- | --- | --- | --- | --- | --- | --- | --- | --- | --- | --- |
|  | **1** | **2** | **3** | **4** | **1a** | **1b** | **1** | **2** | **3** |  |
| Castillo-Martinez 2020 | yes | yes | yes | yes | No - The study does not control for age, sex, and marital status (one star) | yes | yes | yes | yes | 8 |
| Chung 2014 | yes | No- no selection of the non-exposed cohort (not drawn from the same community as the exposed cohort (one star)) | yes | yes | No - The study does not control for age, sex, and marital status (one star) | yes | yes | yes | yes | 7 |
| Chiaranda 2013 | yes | No- no selection of the non-exposed cohort (not drawn from the same community as the exposed cohort (one star)) | yes | yes | No - The study does not control for age, sex and marital status (one star) | yes | yes | yes | No – No Adequacy of follow-up of cohorts a) Complete follow up- all subject accounted for (one star), b) Subjects lost to follow up unlikely to introduce bias- number lost less than or equal to 20% or description of those lost suggested no different from those followed. (one star) | 6 |
| Matsuzawa 2013 | yes | No- no selection of the non-exposed cohort (not drawn from the same community as the exposed cohort (one star)) | yes | yes | No - The study does not control for age, sex, and marital status (one star) | yes | yes | yes | yes | 7 |
| Ozawa 2021 | yes | yes | yes | yes | No - The study does not control for age, sex, and marital status (one star) | yes | yes | yes | yes | 8 |
| Pulignano 2020 | yes | No- no selection of the non-exposed cohort (not drawn from the same community as the exposed cohort (one star)) | yes | yes | No - The study does not control for age, sex, and marital status (one star) | yes | yes | yes | yes | 7 |
| Emami 2018 | yes | yes | yes | yes | No - The study does not control for age, sex and marital status (one star) | yes | No – No assessment of outcome either through a) Independent blind assessment (one star) or b) Record linkage (one star) | No – No follow-up long enough for outcomes to occur (one star) | No – No Adequacy of follow-up of cohorts a) Complete follow up- all subject accounted for (one star) , b)  Subjects lost to follow up unlikely to introduce bias- number lost less than or equal to 20% or description of those lost suggested no different from those followed. (one star) | 5 |
| Katano 2022 | yes | No- no selection of the non-exposed cohort (not drawn from the same community as the exposed cohort (one star)) | yes | yes | No - The study does not control for age, sex, and marital status (one star) | yes | yes | yes | No – No Adequacy of follow-up of cohorts a) Complete follow up- all subject accounted for (one star) , b)  Subjects lost to follow up unlikely to introduce bias- number lost less than or equal to 20% or description of those lost suggested no different from those followed. (one star) | 6 |
| Saito 2022a | yes | yes | yes | yes | No - The study does not control for age, sex, and marital status (one star) | yes | yes | **yes** | yes | 8 |
| Sato 2020 | yes | No- no selection of the non-exposed cohort (not drawn from the same community as the exposed cohort (one star)) | yes | yes | No - The study does not control for age, sex, and marital status (one star) | yes | yes | yes | yes | 7 |
| Tsuchida 2018 | yes | yes | yes | yes | No - The study does not control for age, sex, and marital status (one star) | yes | No – No assessment of outcome either through a) Independent blind assessment (one star) or b) Record linkage (one star) | No – No follow-up long enough for outcomes to occur (one star) | No – No Adequacy of follow-up of cohorts a) Complete follow up- all subject accounted for (one star) , b)  Subjects lost to follow up unlikely to introduce bias- number lost less than or equal to 20% or description of those lost suggested no different from those followed. (one star) | 5 |
| Kitai 2021 | yes | No- no selection of the non-exposed cohort (not drawn from the same community as the exposed cohort (one star)) | yes | yes | No - The study does not control for age, sex, and marital status (one star) | yes | yes | yes | No – No Adequacy of follow-up of cohorts a) Complete follow up- all subject accounted for (one star) , b)  Subjects lost to follow up unlikely to introduce bias- number lost less than or equal to 20% or description of those lost suggested no different from those followed. (one star) | 6 |
| Bieger 2023 | yes | yes | yes | yes | No - The study does not control for age, sex, and marital status (one star) | yes | yes | yes | No – No Adequacy of follow-up of cohorts a) Complete follow up- all subject accounted for (one star) , b)  Subjects lost to follow up unlikely to introduce bias- number lost less than or equal to 20% or description of those lost suggested no different from those followed. (one star) | 7 |
| Eschalier 2021 | yes | yes | yes | yes | No - The study does not control for age, sex, and marital status (one star) | yes | yes | yes | No – No Adequacy of follow-up of cohorts a) Complete follow up- all subject accounted for (one star) , b)  Subjects lost to follow up unlikely to introduce bias- number lost less than or equal to 20% or description of those lost suggested no different from those followed. (one star) | 7 |
| Fonseca 2020 | yes | yes | yes | yes | No - The study does not control for age, sex, and marital status (one star) | yes | No – No assessment of outcome either through a) Independent blind assessment (one star) or b) Record linkage (one star) | No – No follow-up long enough for outcomes to occur (one star) | No – No Adequacy of follow-up of cohorts a) Complete follow up- all subject accounted for (one star) , b)  Subjects lost to follow up unlikely to introduce bias- number lost less than or equal to 20% or description of those lost suggested no different from those followed. (one star) | 5 |
| Saito 2022b | yes | yes | yes | yes | No - The study does not control for age, sex, and marital status (one star) | yes | yes | **yes** | yes | 8 |
| Kono 2020 | yes | yes | yes | yes | No - The study does not control for age, sex, and marital status (one star) | yes | No – No assessment of outcome either through a) Independent blind assessment (one star) or b) Record linkage (one star) | No – No follow-up long enough for outcomes to occur (one star) | No – No Adequacy of follow-up of cohorts a) Complete follow up- all subject accounted for (one star) , b)  Subjects lost to follow up unlikely to introduce bias- number lost less than or equal to 20% or description of those lost suggested no different from those followed. (one star) | 5 |
| Ogawa 2020 | yes | yes | yes | yes | No - The study does not control for age, sex, and marital status (one star) | yes | No – No assessment of outcome either through a) Independent blind assessment (one star) or b) Record linkage (one star) | No – No follow-up long enough for outcomes to occur (one star) | No – No Adequacy of follow-up of cohorts a) Complete follow up- all subject accounted for (one star) , b)  Subjects lost to follow up unlikely to introduce bias- number lost less than or equal to 20% or description of those lost suggested no different from those followed. (one star) | 5 |
| Onoue 2016 | yes | yes | yes | yes | No - The study does not control for age, sex, and marital status (one star) | yes | yes | yes | No – No Adequacy of follow-up of cohorts a) Complete follow up- all subject accounted for (one star) , b)  Subjects lost to follow up unlikely to introduce bias- number lost less than or equal to 20% or description of those lost suggested no different from those followed. (one star) | 7 |
| Peng 2023 | yes | yes | yes | yes | No - The study does not control for age, sex, and marital status (one star) | yes | No – No assessment of outcome either through a) Independent blind assessment (one star) or b) Record linkage (one star) | No – No follow-up long enough for outcomes to occur (one star) | No – No Adequacy of follow-up of cohorts a) Complete follow up- all subject accounted for (one star) , b)  Subjects lost to follow up unlikely to introduce bias- number lost less than or equal to 20% or description of those lost suggested no different from those followed. (one star) | 5 |
| Valdiviesso 2022 | yes | yes | yes | yes | No - The study does not control for age, sex, and marital status (one star) | yes | No – No assessment of outcome either through a) Independent blind assessment (one star) or b) Record linkage (one star) | No – No follow-up long enough for outcomes to occur (one star) | No – No Adequacy of follow-up of cohorts a) Complete follow up- all subject accounted for (one star) , b)  Subjects lost to follow up unlikely to introduce bias- number lost less than or equal to 20% or description of those lost suggested no different from those followed. (one star) | 5 |
| Harada 2017 | yes | yes | yes | yes | No - The study does not control for age, sex, and marital status (one star) | yes | No – No assessment of outcome either through a) Independent blind assessment (one star) or b) Record linkage (one star) | No – No follow-up long enough for outcomes to occur (one star) | No – No Adequacy of follow-up of cohorts a) Complete follow up- all subject accounted for (one star), b)  Subjects lost to follow up unlikely to introduce bias- number lost less than or equal to 20% or description of those lost suggested no different from those followed. (one star) | 5 |
