# Supplementary figures and images for: Is sarcopenia an associated factor of increased administration of specific medications in patients with heart failure? A systematic review and meta-analysis
Source: Front Cardiovasc Med. 2024 Jan 25;11:1293537. doi: 10.3389/fcvm.2024.1293537 (PMC10850377; doi:10.3389/fcvm.2024.1293537)

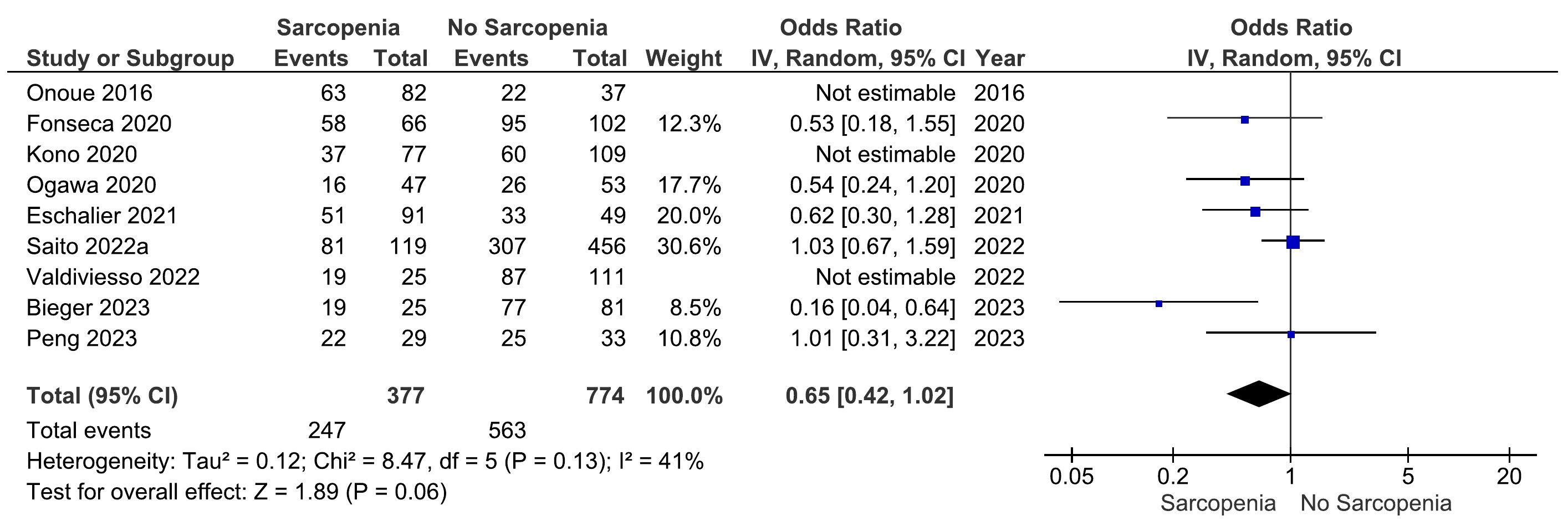

Supplement: Supplementary Figure S1 — Association of ACE-I/ARB administration in patients with HF and sarcopenia versus without sarcopenia according to sarcopenia definition. [file Image1.jpeg]

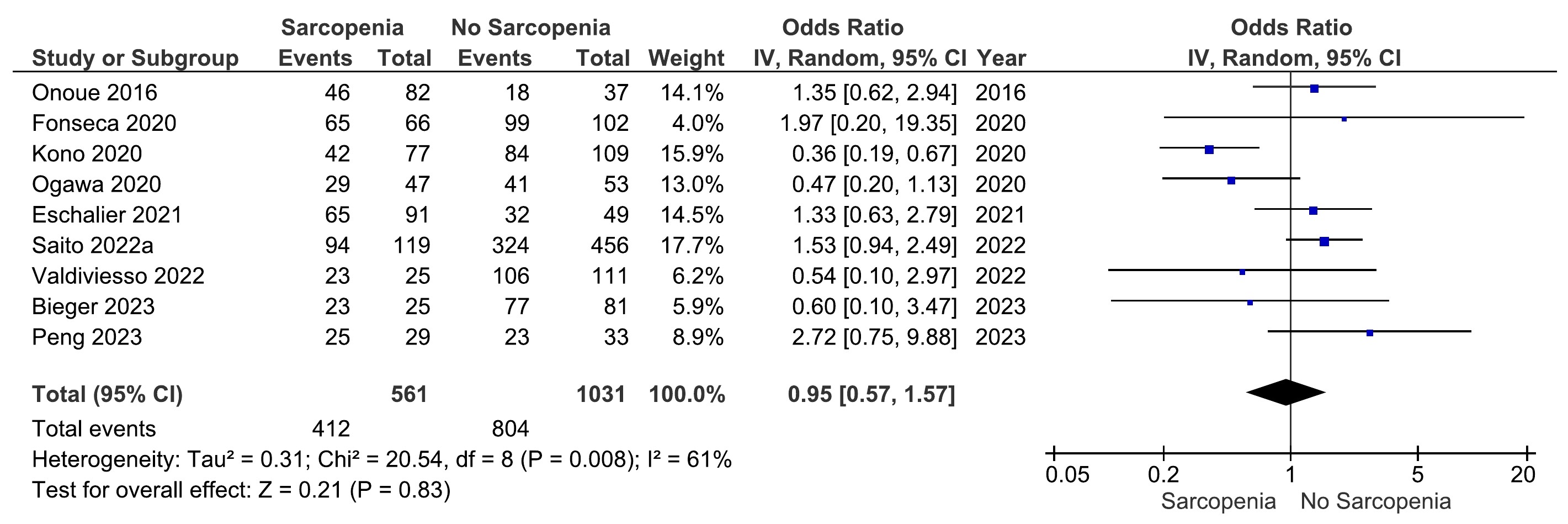

Supplement: Supplementary Figure S2 — Association of B-blocker administration in patients with HF and sarcopenia versus without sarcopenia according to sarcopenia definition. [file Image2.jpeg]

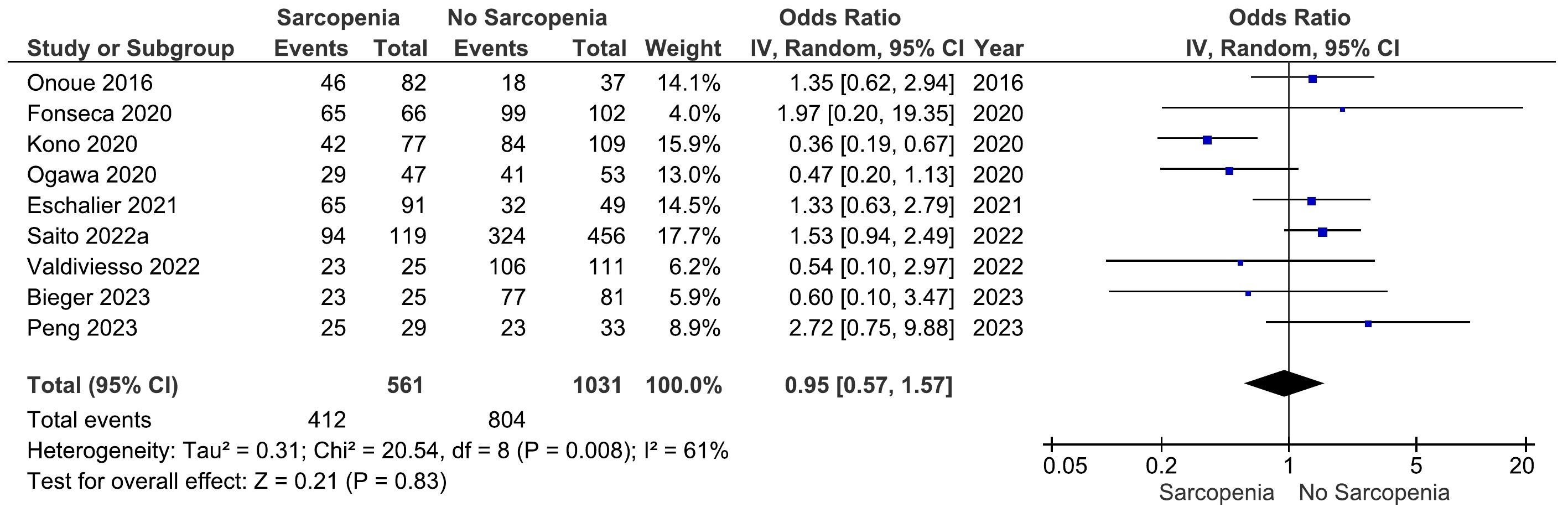

Supplement: Supplementary Figure S3 — Association of loop diuretic administration in patients with HF and sarcopenia versus without sarcopenia according to sarcopenia definition. [file Image3.jpeg]

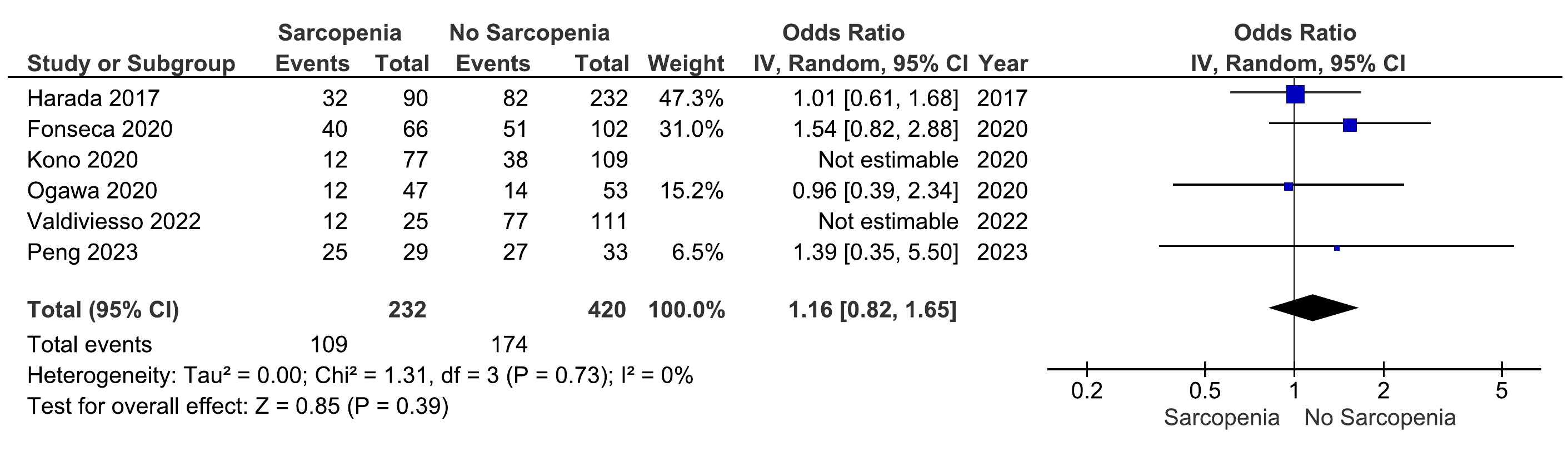

Supplement: Supplementary Figure S4 — Association of statin administration in patients with HF and sarcopenia versus without sarcopenia according to sarcopenia definition. [file Image4.jpeg]

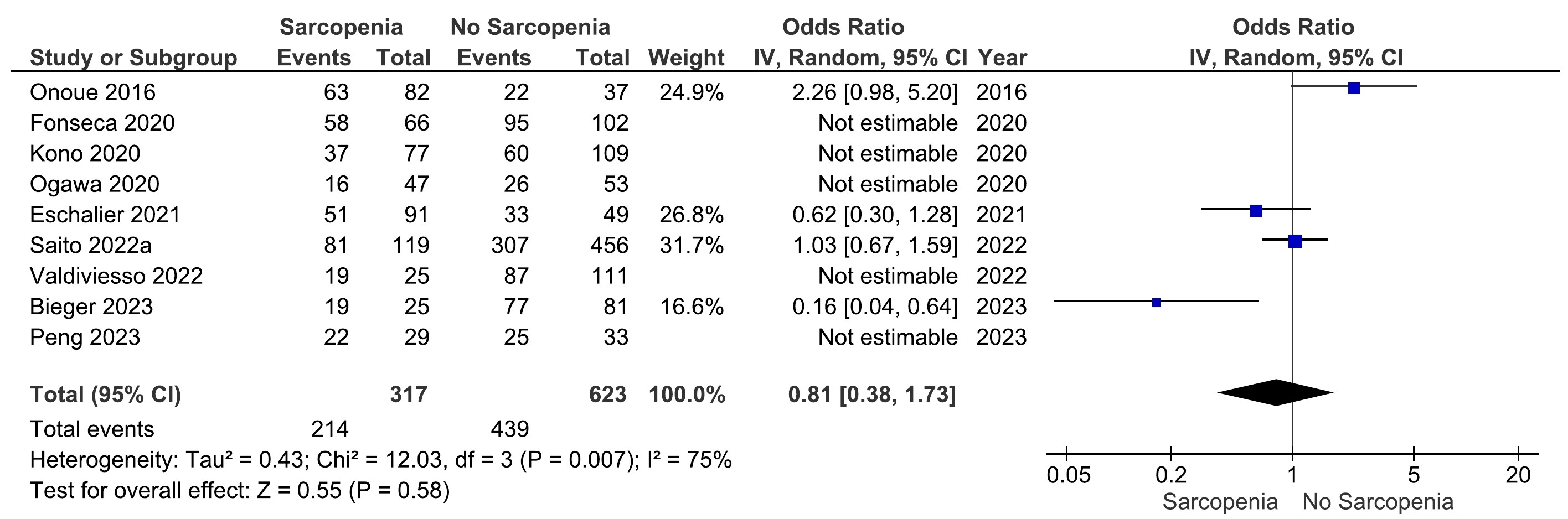

Supplement: Supplementary Figure S5 — Association of ACE-I/ARB administration in patients with HF and sarcopenia versus without sarcopenia based on risk of bias. [file Image5.jpeg]

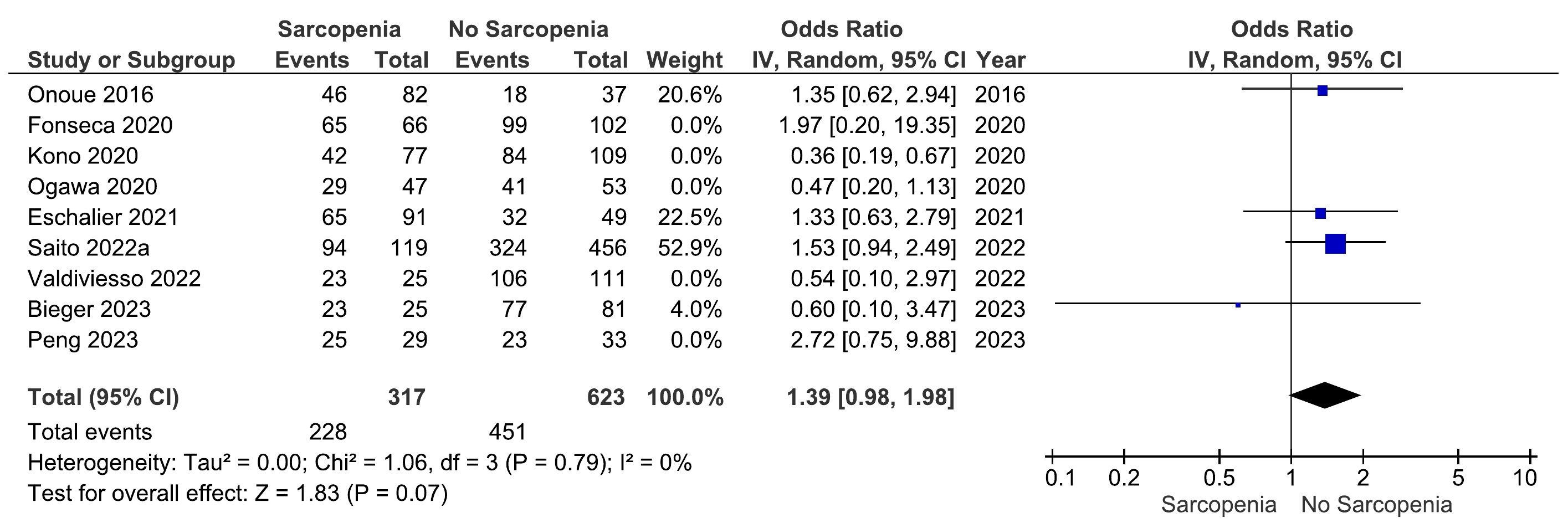

Supplement: Supplementary Figure S6 — Association of B-blockers administration in patients with HF and sarcopenia versus without sarcopenia based on risk of bias. [file Image6.jpeg]

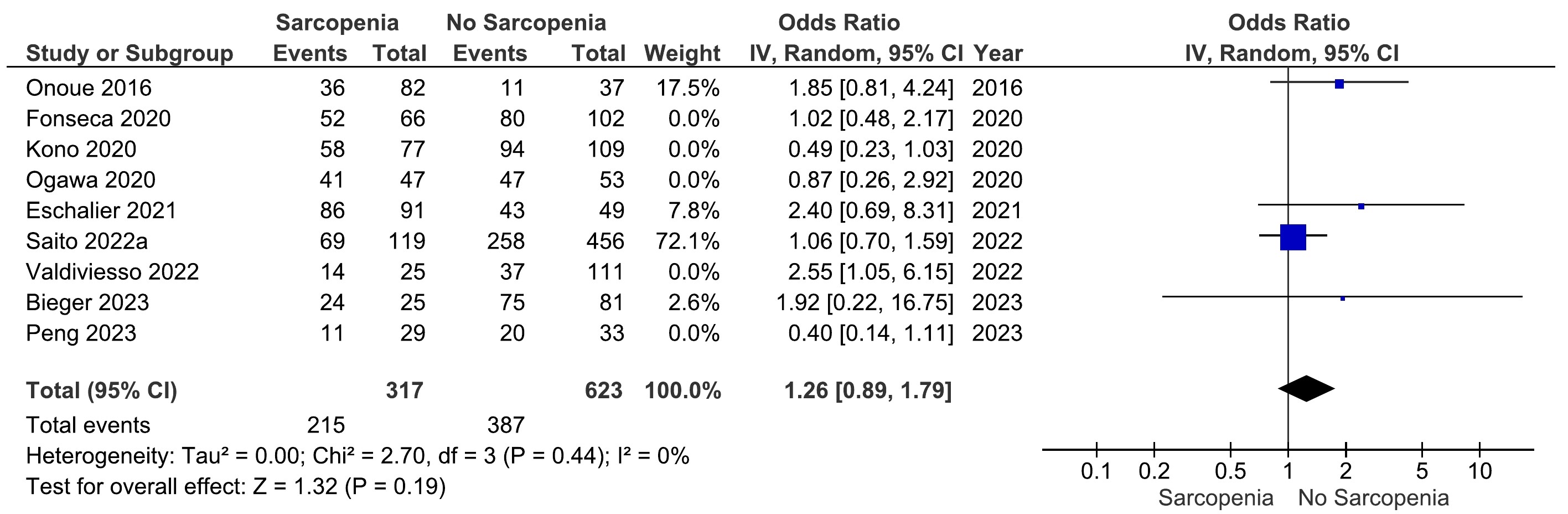

Supplement: Supplementary Figure S7 — Association of loop diuretic administration in patients with HF and sarcopenia versus without sarcopenia based on risk of bias. [file Image7.jpeg]

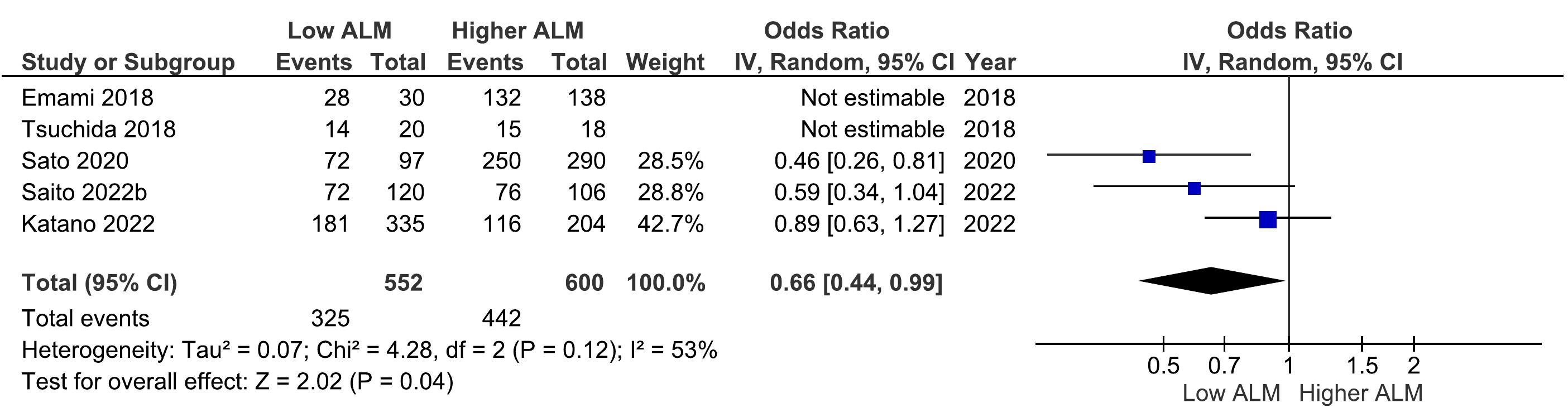

Supplement: Supplementary Figure S8 — Association of ACE-I/ARB administration in patients with HF and higher ALM versus low ALM based on risk of bias. [file Image8.jpeg]

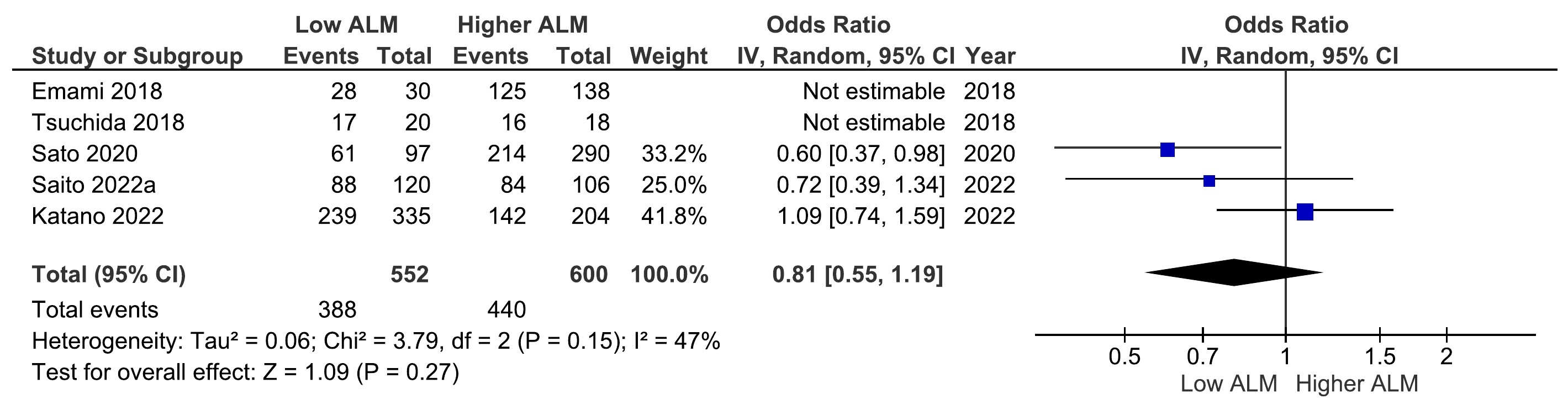

Supplement: Supplementary Figure S9 — Association of B-blockers administration in patients with HF and higher ALM versus low ALM based on risk of bias. [file Image9.jpeg]
